# Diazotrophic community in the sediments of Poyang Lake in response to water level fluctuations

**DOI:** 10.3389/fmicb.2024.1324313

**Published:** 2024-02-02

**Authors:** Qiang Wu, Fei Wang, Yuwei Chen, Wenxiang Zou, Zhigang Zhu

**Affiliations:** ^1^School of Hydraulic and Ecological Engineering, Nanchang Institute of Technology, Nanchang, China; ^2^Jiangxi Key Laboratory of Poyang Lake Water Resources and Environment, Jiangxi Academy of Water Science and Engineering, Nanchang, China; ^3^Jiangxi Provincial Technology Innovation Center for Ecological Water Engineering in Poyang Lake Basin, Nanchang, China

**Keywords:** diazotrophic community, water level fluctuations, floodplain lake, sediment, co-occurrence network

## Abstract

Water level fluctuations (WLFs) are typical characteristic of floodplain lakes and dominant forces regulating the structure and function of lacustrine ecosystems. The sediment diazotrophs play important roles in contributing bioavailable nitrogen to the aquatic environment. However, the relationship between the diazotrophic community and WLFs in floodplain lakes is unknown. In this paper, we carried out a comprehensive investigation on the alpha diversity, abundance, composition and co-occurrence network of the sediment diazotrophs during different water level phases in Poyang Lake. There were no regular variation patterns in the alpha diversity and abundance of the sediment diazotrophs with the water level phase transitions. The relative abundance of some diazotrophic phyla (including *Alphaproteobacteria*, *Deltaproteobacteri*, *Euryarchaeota*, and *Firmicutes*) and genera (including *Geobacter*, *Deferrisoma*, *Desulfuromonas*, *Rivicola*, *Paraburkholderia*, *Methylophilus*, *Methanothrix*, *Methanobacterium*, and *Clostridium*) was found to change with the water level phase transitions. The results of ANOSIM, PerMANOVA, and DCA at the OTU level showed that the diazotrophic community structure in the low water level phase was significantly different from that in the two high water level phases, while there was no significant difference between the two high water level phases. These results indicated that the diazotrophic community was affected by the declining water level in terms of the composition, while the rising water level contributed to the recoveries of the diazotrophic community. The diazotrophs co-occurrence network was disrupted by the declining water level, but it was strengthened by the rising water level. Moreover, redundancy analysis showed that the variation of the diazotrophic community composition was mostly related to sediment total nitrogen (TN) and total phosphorous (TP). Interestingly, the levels of sediment TN and TP were also found to vary with the water level phase transitions. Therefore, it might be speculated that the WLFs may influence the sediment TN and TP, and in turn influence the diazotrophic community composition. These data can contribute to broadening our understanding of the ecological impacts of WLFs and the nitrogen fixation process in floodplain lakes.

## 1 Introduction

Biological nitrogen fixation, a microbial process that converts the reduction of dinitrogen gas (N_2_) to biologically available ammonium, plays a fundamental role in the primary production of aquatic systems (Vitousek and Howarth, 1991; Arp, 2000; Montoya et al., 2004). Diazotrophs in the aquatic environment are widely distributed among *Proteobacteria*, *Euryarchaeota*, *Cyanobacteria*, *Bacteroidetes*, and *Firmicutes* based on the diversity of the *nifH* gene (Kapili et al., 2020; Wang et al., 2020; Tian et al., 2021a). Diazotrophs capable of fixing molecular nitrogen have been found to be present in the water columns and the sediments of lakes (Zhang and Zhang, 2016; Yao et al., 2018; Wang et al., 2020; Tian et al., 2021a). It is traditionally believed that N_2_-fixing *Cyanobacteria* are the main diazotrophs in aquatic systems (Yao et al., 2018). However, nitrogen fixation by heterotrophic diazotrophs in benthic sediments also contributes a certain amount of bioavailable nitrogen to the aquatic environment (Yao et al., 2018). In the Lake Waco Wetland, near Waco, Texas, United States, the contribution of sediment nitrogen fixation to the N budget can support sustained nitrogen-cycling processes such as denitrification and dissimilatory NO_3_^−^ reduction to ammonium when overlying water inorganic N supply rates are low (Scott et al., 2008). In Lake Taihu, the annual amount of N_2_ fixed in the sediments is much higher than that fixed in the water column and offsets about 1.8% of N_2_ loss through denitrification (Yao et al., 2018). Previous studies have suggested that the relative abundance of genes involved in nitrogen fixation in sediment microbial community is higher compared with its corresponding water microbial community in the lake ecosystem (Ren et al., 2019a, 2023).

Water level fluctuations (WLFs), an important component of hydrodynamics, are typical characteristic of floodplain lakes and dominant forces regulating the structure and function of lacustrine ecosystems (Leira and Cantonati, 2008; Wantzen et al., 2008; Wang et al., 2011; Evtimova and Donohue, 2016). It has been widely reported that WLFs have an impact on the physical, chemical, and biological properties of lake ecosystems. WLFs can alter lake light conditions, water temperature, sedimentation and morphometry in terms of physical properties (Coops et al., 2003; Hofmann et al., 2008; Liu et al., 2016). The dynamics of water quality characteristics and sediment nutrient release in floodplain lakes can be regulated by WLFs (Ni et al., 2015; Liu et al., 2016; Wang S. Y. et al., 2021; Geng et al., 2022). The community of macrophytes, phytoplankton, microorganisms and benthic invertebrates has been shown to change with WLFs in natural lakes (Coops et al., 2003; Evtimova and Donohue, 2016; Liu et al., 2019; Ren et al., 2019b). However, to our knowledge, no study has illustrated the relationship between the diazotrophic community and WLFs in floodplain lakes. As mentioned above, WLFs can regulate the release of sediment nutrients in floodplain lakes, and in turn change the nutrient content. Nutrient availability can shape the diazotrophic community in aquatic ecosystems (Weber et al., 2017; Wang et al., 2020; Luo et al., 2021; Tian et al., 2021a; Jabir et al., 2021b). Therefore, elucidating the response of the sediment diazotrophic community to WLFs is of great interest and importance to gain insights into the ecological impacts of WLFs and the nitrogen-cycling processes of the floodplain lake.

Poyang Lake, which is located in one of the most frequently affected areas by a variety of flood or drought events in China, experiences dramatic intra-annual WLFs and its water level ranges from 7.9 to 19.4 m (Li et al., 2013; Liu et al., 2015). In the present study, we carried out a comprehensive investigation on the alpha diversity, abundance, composition and co-occurrence network of the sediment diazotrophs during different hydrological periods (i.e., water level phases) in Poyang Lake. The response mechanism of the sediment diazotrophic community to WLFs in this lake is then discussed where possible.

## 2 Materials and methods

### 2.1 Study area and sample collection

Poyang Lake (28°24′–29°46′ N; 115° 49′–116°46′ E), the largest freshwater lake in China, is located on the south bank of the Yangtze River in the northern part of Jiangxi Province. The basin area of the lake is 1.62 × 10^5^ km^2^. The lake is mainly fed by five tributaries (Gan River, Fu River, Xin River, Rao River and Xiu River) in Jiangxi Province and provides regulation and storage capacity prior to their intersection with the Yangtze River through an estuary (Wang and Liang, 2015). Poyang Lake is regulated both by the five tributaries and the Yangtze River, which leads to its intra-annual fluctuation of water level between the wet season and the dry season (Zhao et al., 2011). The lake’s highest water level is usually between 18 and 21 m above mean sea level during the summer rainy season, and the lowest level is usually between 8.8 and 11.4 m above mean sea level during the autumn or winter dry season (Liu et al., 2019). Generally, flooding occurs during the wet season from April to October, while during the dry season from November to March, about 90% of the lake’s water is lost, exposing vast areas of grassy marshland (Yang et al., 2015). Poyang Lake wetland is rich in biodiversity, and has been recognized by the UN as one of the world’s important wetlands (Zhang et al., 2012).

**Figure 1:**
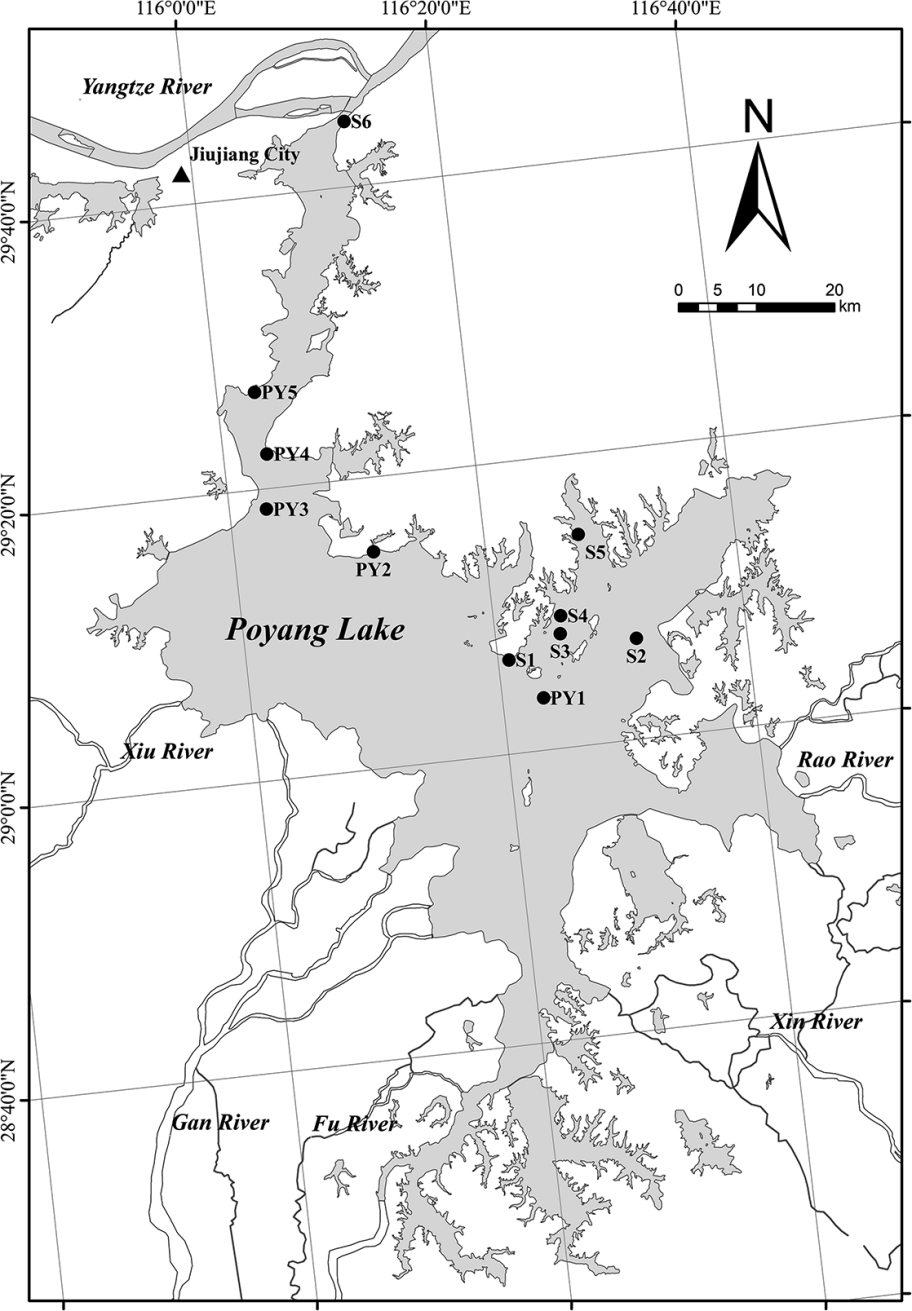
Study area and sampling sites (black dots) in Poyang Lake, China.

High and low water level phases of Poyang Lake are defined by days when the water level at the Xingzi station is above or below 14 m (Liu et al., 2015). Water level data for the period January 2021 to December 2022 were available on the hydrological monitoring center of the Jiangxi Province websites.^1^ The two high water level periods were therefore 2 May to 24 October 2021 and 29 April to 28 July 2022. We collected five primary sediment samples (PY1-PY5) from the first high water level phase (19 October 2021, 14.9 m a.s.l, referred to as HWL-2021), the low water level phase (3 January 2022, 7.8 m a.s.l, referred to as LWL-2022) and the second high water level phase (20 May 2022, 16.1 m a.s.l, referred to as HWL-2022), respectively (Figure 1). Moreover, three (S1, S2, and S4) and four (S2, S3, S5, and S6) supplementary samples were collected at HWL-2021 and HWL-2022, respectively (Figure 1). We could not collect samples from these supplementary sites at other sampling times, either because of low water levels or because the sampler did not collect sediments. Surface sediments (0–10 cm) were collected in triplicate using a Peterson’s grab sampler at each site. Triplicate subsamples from each site were mixed together for homogenization. The sediment samples were placed in sealable plastic bags, stored in a cooler box and then transported to the laboratory for analysis. Each sample was divided into two parts, including one for physicochemical analysis, and another kept frozen at −40°C for the diazotrophic community analysis.

### 2.2 Physicochemical analysis

Sediment pH and electrical conductivity (EC) were, respectively, determined using a pH meter (FE20, Mettler Toledo, Switzerland) and a benchtop conductivity meter (Orion 3-Star, Thermo, United States). Sediment organic matter (OM) content was determined using the method of Yao et al. (2018). The total nitrogen (TN) and total phosphorous (TP) contents of the sediments were, respectively, measured using the alkaline potassium persulfate spectrophotometric method (Kjeldahl, 1883) and molybdenum blue colorimetry method (Jin and Tu, 1990) after passing air-dried sediments through a standard sieve (0.15 mm).

### 2.3 DNA extraction, Illumina MiSeq sequencing and diazotrophic quantification

Microbial DNA was extracted from sediment samples (~0.4 g) using FastDNA SPIN Kit for Soil (MP Biomedicals, United States) according to the manufacturer protocol. The *nifH* gene fragments of the diazotrophic community were amplified with the barcode primers polF (5′-TGYGAYCCNAARGCNGA-3′) and polR (5′-ADNGCCATCATYTCNCC-3′) (Zehr and McReynolds, 1989). The amplification program included an initial denaturation step of 95°C for 5 min, followed by 34 cycles at a melting temperature of 95°C for 30 s, an annealing temperature of 55°C for 30 s, and an extension temperature of 72°C for 45 s, and finally a final extension step of 72°C for 10 min. Triplicate PCR products for each sample were pooled and then verified on a 2.0% agarose gel. Amplicons were purified using AxyPrep DNA Gel Extraction Kit (Axygen, United States), and quantified using QuantiFluor^™^-ST (Promega, USA). The purified PCR products were mixed in equimolar ratios, and sequenced (2 × 250 bases, paired-end) on an Illumina MiSeq platform (Illumina, United States) following the manufacturer’s protocols. Raw sequences from this study were deposited in the NCBI Sequence Read Archive (SRA) under accession number PRJNA1022843.

The diazotrophic community abundance was assessed using quantitative PCR (qPCR) assay with the abovementioned primers polF and polR. Amplification was performed in triplicate in 25 μL reaction mixture that contained 2 μL template DNA, 12.5 μL qPCR Mix (2×, TaKara) and 1 μL of each primer (10 μM). Standard curve was constructed by tenfold serial dilution of known amounts of plasmids containing target *nifH* gene fragments. To exclude any possible contamination, negative control containing no template DNA was carried out in each experiment.

### 2.4 Data processing and analyses

Raw sequences from the Illumina MiSeq sequencing were quality-controlled using Trimmomatic (Bolger et al., 2014) and merged using FLASH^2^ according to the criteria described in Yuan et al. (2015). The trimmed sequences were clustered at 97% identity into operational taxonomic units (OTUs) using UPARSE (version 7.1^3^). Representative sequences in the OTUs were aligned against NCBI database to identify species information (Wu et al., 2020). The number of qualified reads ranged from 30,868 to 57,242 in all samples. To correct the differences in sequencing depth, each sample was randomly rarefied to 30,868 reads. The alpha diversity indices (OTU Richness, Chao richness estimators and Shannon diversity index) and the rarefaction curve of the diazotrophic community were analyzed using mothur (Schloss et al., 2009).

Linear and quadratic models were used to explore the relationships between the alpha diversity indices and the abundance of the diazotrophic community by the *trendline* function in the R package basicTrendline (Mei et al., 2020). The Canoco 5 software (Microcomputer Power, United States) was used to compare the composition of diazotrophic communities between samples at the OTU level. Detrended correspondence analyses (DCA) was suggested by the Canoco 5 program because the longest gradient length was found to be >3 during the course of the analysis. To evaluate significant differences in the diazotrophic communities between different water level phases, two-way crossed analysis of similarities (ANOSIM) and permutational multivariate analysis of variance (PerMANOVA) were performed in the vegan package with R software. To elucidate network interactions in the diazotrophic communities, the RMT-based molecular ecological network analysis were performed using the Molecular Ecological Network Analyses pipeline^4^ (Deng et al., 2012). The constructed ecological networks were then visualized by using Gephi (Bastian et al., 2009). To recognize the putative keystone taxa, the connectivity of each node was determined based on both its within-module connectivity (Zi) and among-module connectivity (Pi) (Guimera and Amaral, 2005).

Correlations between diazotrophic communities and environmental factors were detected with redundancy analysis (RDA) using Canoco 5 program. All data were log (*x* + 1) transformed except pH and the redundant environmental variables with VIF > 20 were eliminated before the RDA. The significance of the relationships of environmental variables to the variation in diazotrophic community composition was tested using Monte Carlo permutation tests (499 unrestricted permutations, *p* < 0.05) (Wang et al., 2012). We selected the significant variables (*p* < 0.05) at the forward selection step for further analyses. The relationships between the relative abundance of the abundant diazotrophic phyla or genera (average relative abundance >1%) and the environmental variables were examined with Pearson correlation analysis using the software IBM SPSS Statistics (version 19, IBM Company, United States).

## 3 Results

### 3.1 Changes in sediment characteristics

Table 1 shows the mean sediment property values in the three water level phases. The levels of sediment TN, TP and N:P molar ratio were all significantly (*p* < 0.05) higher in HWL-2021 and HWL-2022 than in LWL-2022. The EC was significantly (*p* < 0.05) lower in HWL-2022 (46.0 ± 34.9 μS cm^−1^) than in HWL-2021 (92.2 ± 58.9 μS cm^−1^) and LWL-2022 (120.1 ± 29.2 μS cm^−1^). The OM content was significantly (*p* < 0.05) higher in HWL-2022 (8.03 ± 0.96%) than in HWL-2021 (6.05 ± 2.01%).

**Table 1:** Sediment properties summarized as means (±standard deviations) in the three water level phases in Poyang Lake.

Parameter	Water level phases		
	HWL-2021	LWL-2022	HWL-2022
pH	6.18 (±0.67)^a^	6.57 (±0.86)^a^	6.82 (±0.50)^a^
EC (μS cm^−1^)	92.2 (±58.9)^a^	120.1 (±29.2)^a^	46.0 (±34.9)^b^
TN (mg kg^−1^)	1975.78 (±371.25)^a^	635.75 (±243.09)^b^	1827.22 (±426.26)^a^
TP (mg kg^−1^)	383.59 (±86.12)^a^	204.25 (±53.85)^b^	409.17 (±100.20)^a^
OM (%)	6.05 (±2.01)^b^	6.53 (±1.14)^ab^	8.03 (±0.96)^a^
N:P (molar ratio)	11.71 (±2.22)^a^	6.79 (±1.16)^b^	10.05 (±2.02)^a^

Different letters (a and b) denote significant differences (a > b, one-way ANOVA).

**Figure 2:**
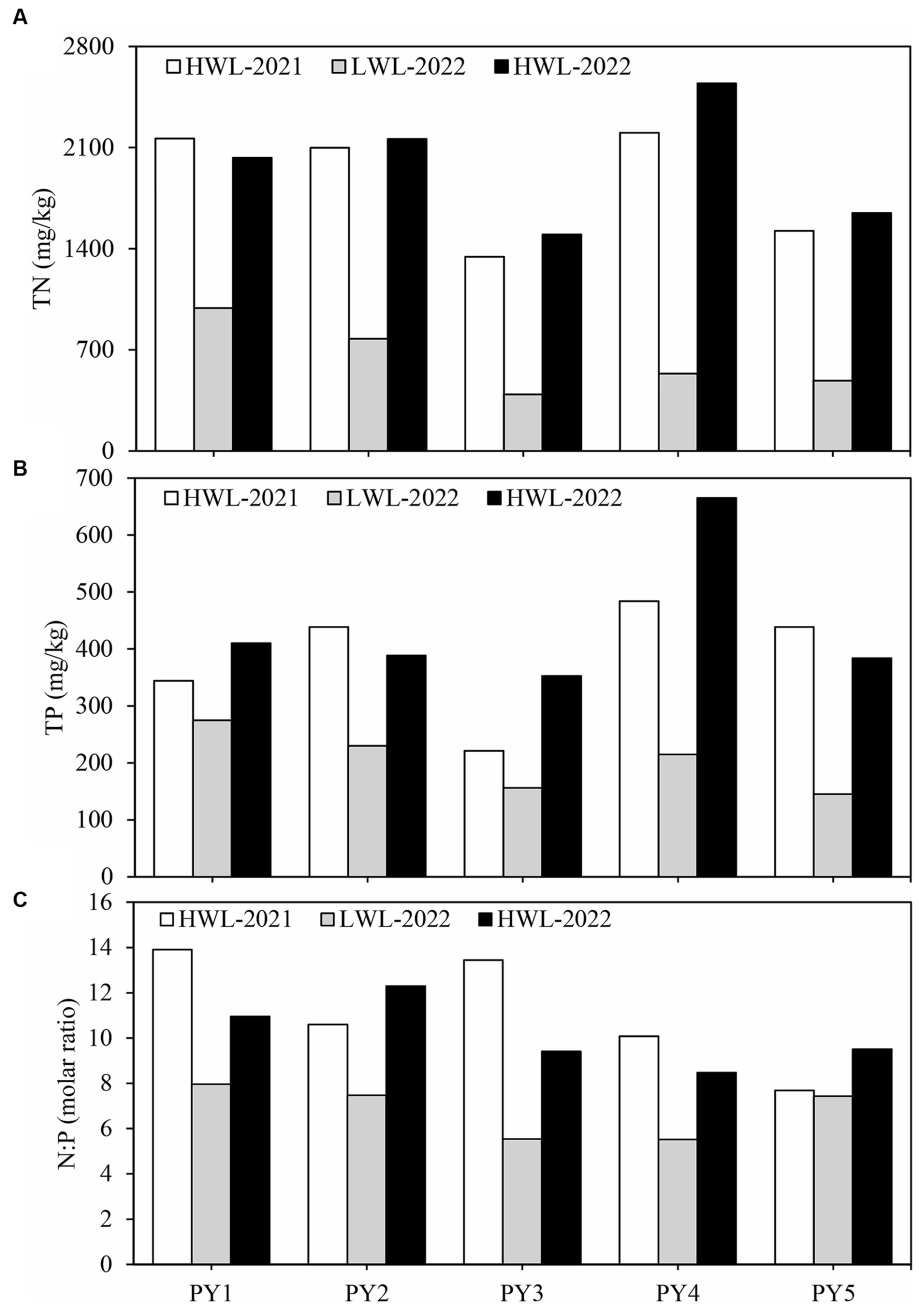
Dynamics of **(A)** TN, **(B)** TP, and **(C)** N:P molar ratio at the five primary sites (PY1-PY5) among different water level phases.

Regular changes in some sediment properties with the water level phase transitions were observed at the five primary sites (PY1-PY5) (Figure 2). The levels of sediment TN, TP and N:P molar ratio were decreased from the first high to low water level phase, and increased from the low to second high water level phase. For the five primary sites, the TN content in HWL-2021 and HWL-2022 was 2.2–4.1 and 2.1–4.7 times that in LWL-2022, respectively. The TP content in HWL-2021 and HWL-2022 was 1.3–3.0 and 1.5–3.1 times that in LWL-2022, respectively. Interestingly, we found no regular variation patterns in the levels of sediment TN, TP and N:P molar ratio at the five primary sites between the two high water level phases (Figure 2).

### 3.2 Diversity and abundance of diazotrophs

Across all diazotrophic community samples in the three water level phases, we obtained 883,739 high-quality sequences. The rarefaction curves of OTUs and Shannon diversity index for each sample reached the plateau and saturation level, respectively (Supplementary Figure S1), suggesting that each diazotrophic community was well represented at the sequencing depth used in the present study. The mean values of the alpha diversity indices and the abundance of diazotrophs in the three water level phases are shown in Supplementary Table S1. The OTU Richness, Chao richness estimators, Shannon diversity index and *nifH* gene of diazotrophs in each water level phase were in the range of 2089 ± 841 to 2,402 ± 1,344, 2,550 ± 987 to 2,810 ± 1,610, 6.38 ± 0.92 to 6.62 ± 0.51 and 2.80 ± 1.38 × 10^8^ to 6.63 ± 4.17 × 10^8^ copies g^−1^, respectively. We did not observe regular variation patterns in the alpha diversity indices and the abundance of diazotrophs at the five primary sites with the water level phase transitions (Supplementary Figure S2). Moreover, the abundance of diazotrophs was significantly and positively correlated with OTU Richness (*R*^2^ = 0.37, *p* < 0.01) (Supplementary Figure S3A), Chao richness estimators (*R*^2^ = 0.34, *p* < 0.01) (Supplementary Figure S3B) and Shannon diversity index (*R*^2^ = 0.28, *p* < 0.05) (Supplementary Figure S3C). The Shannon diversity index was significantly and negatively correlated with pH (Supplementary Table S2). The abundance of diazotrophs was significantly and positively related to OM (Supplementary Table S2).

### 3.3 Changes in diazotrophic community composition

**Figure 3:**
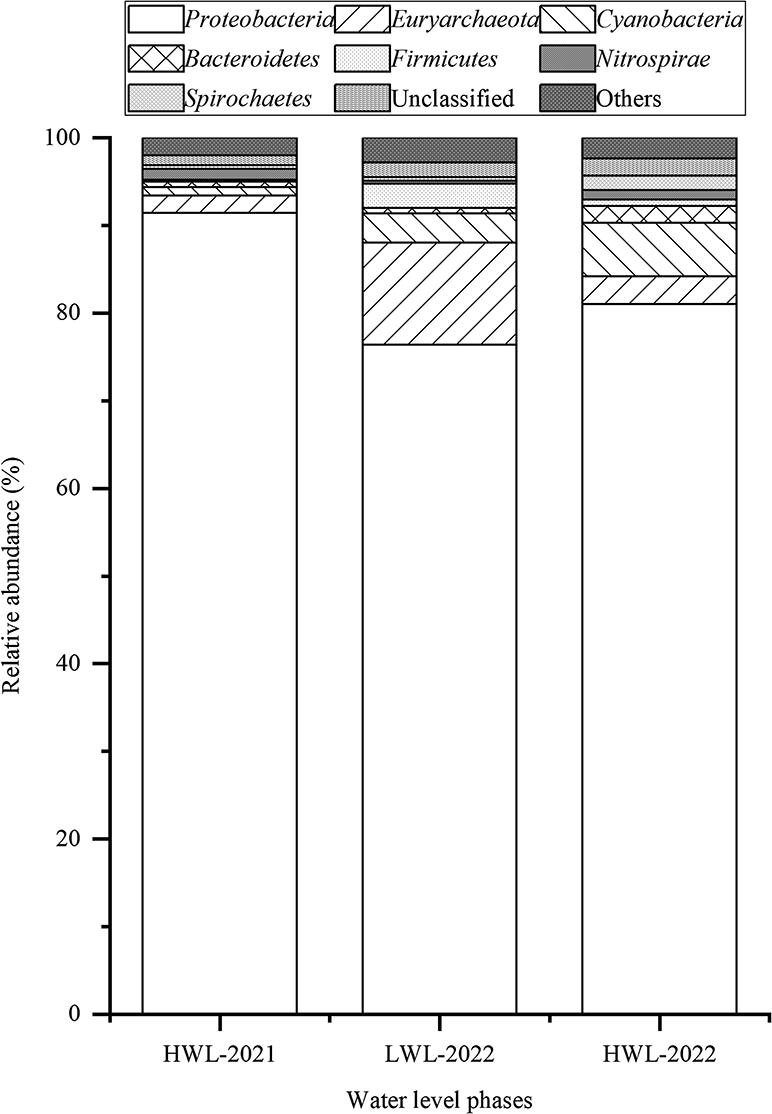
Relative abundance of the diazotrophic phyla found in the three water level phases. The phyla at a relative abundance of <1% were included in others.

**Figure 4:**
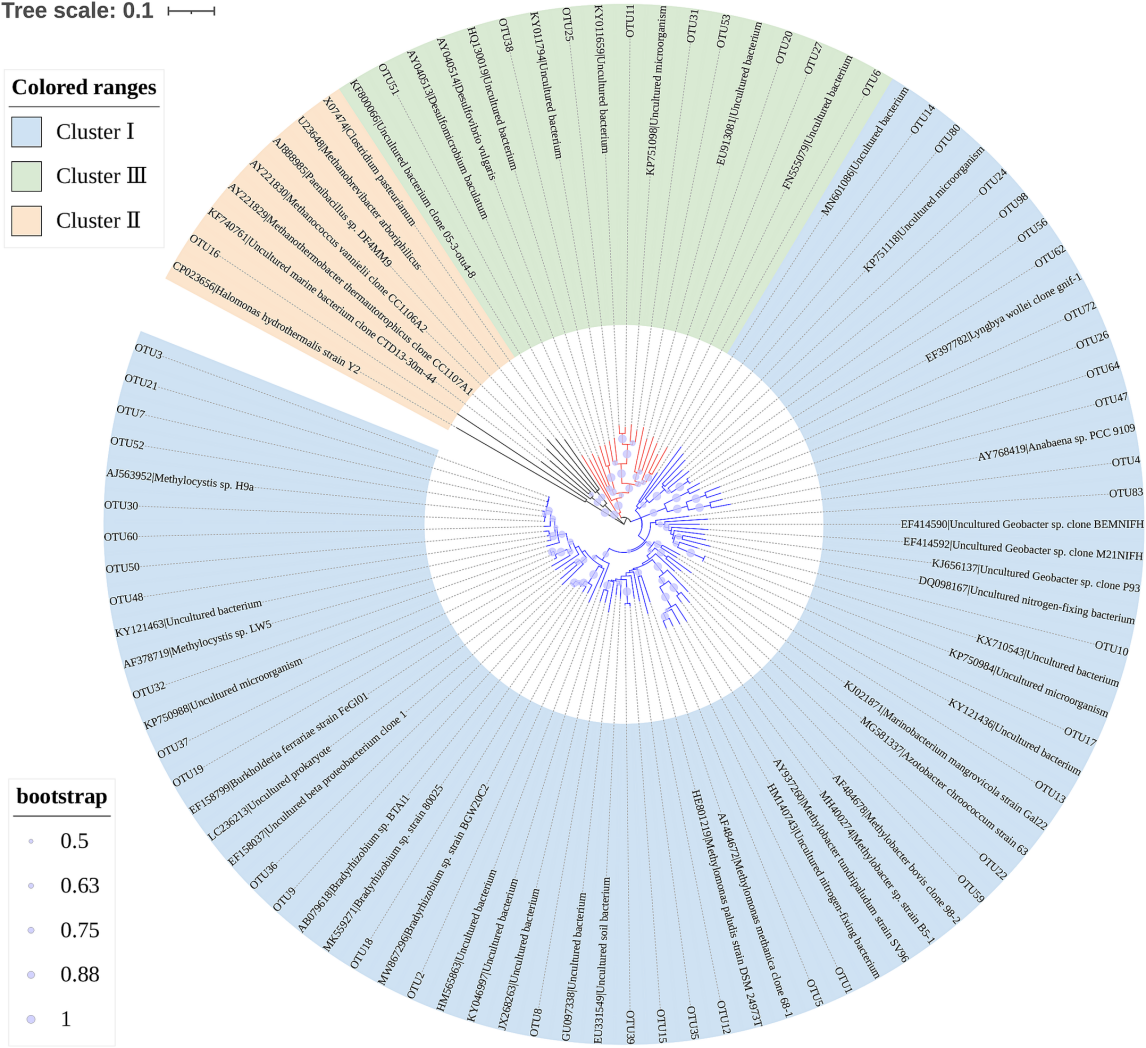
Neighbor-joining phylogenetic tree of the *nifH* sequences for representative OTUs (relative abundance >0.2% of total sequences) derived from the sediments of Poyang Lake. Bootstrap values (1,000 replicates) > 50% are indicated with size proportional purple circles. This tree was visualized by using iTOL (https://itol.embl.de/) (Letunic and Bork, 2007).

Figure 3 reveals that *Proteobacteria*, *Euryarchaeota*, *Cyanobacteria*, *Bacteroidetes*, *Firmicutes*, *Nitrospirae*, and *Spirochaetes* were the main diazotrophs in the sediments of Poyang Lake. These phyla accounted for 95.5–96.8% of the sequences in each water level phase. *Proteobacteria* was the most dominant diazotrophic phylum, and constituted 76.4–91.4% of the total sequences in each water level phase. Additionally, *Alphaproteobacteria*, *Betaproteobacteria*, *Deltaproteobacteria* and *Gammaproteobacteria* were the four dominant classes within the members of *Proteobacteria*, accounting for >99.6% of the total sequences belonging to *Proteobacteria* in each water level phase (Supplementary Figure S4). Diazotrophs are generally clustered into four basic groups (Cluster I, II, III and IV) based on phylogenetic distribution of *nifH* genes (Chien and Zinder, 1996). In this study, 49 *nifH* sequences of representative OTUs (relative abundance >0.2% of total sequences) were selected to construct the phylogenetic tree. All of the selected OTUs were grouped into three clusters, Cluster I (39/49), Cluster III (9/49) and Cluster II diazotrophs (1/49) (Figure 4).

**Figure 5:**
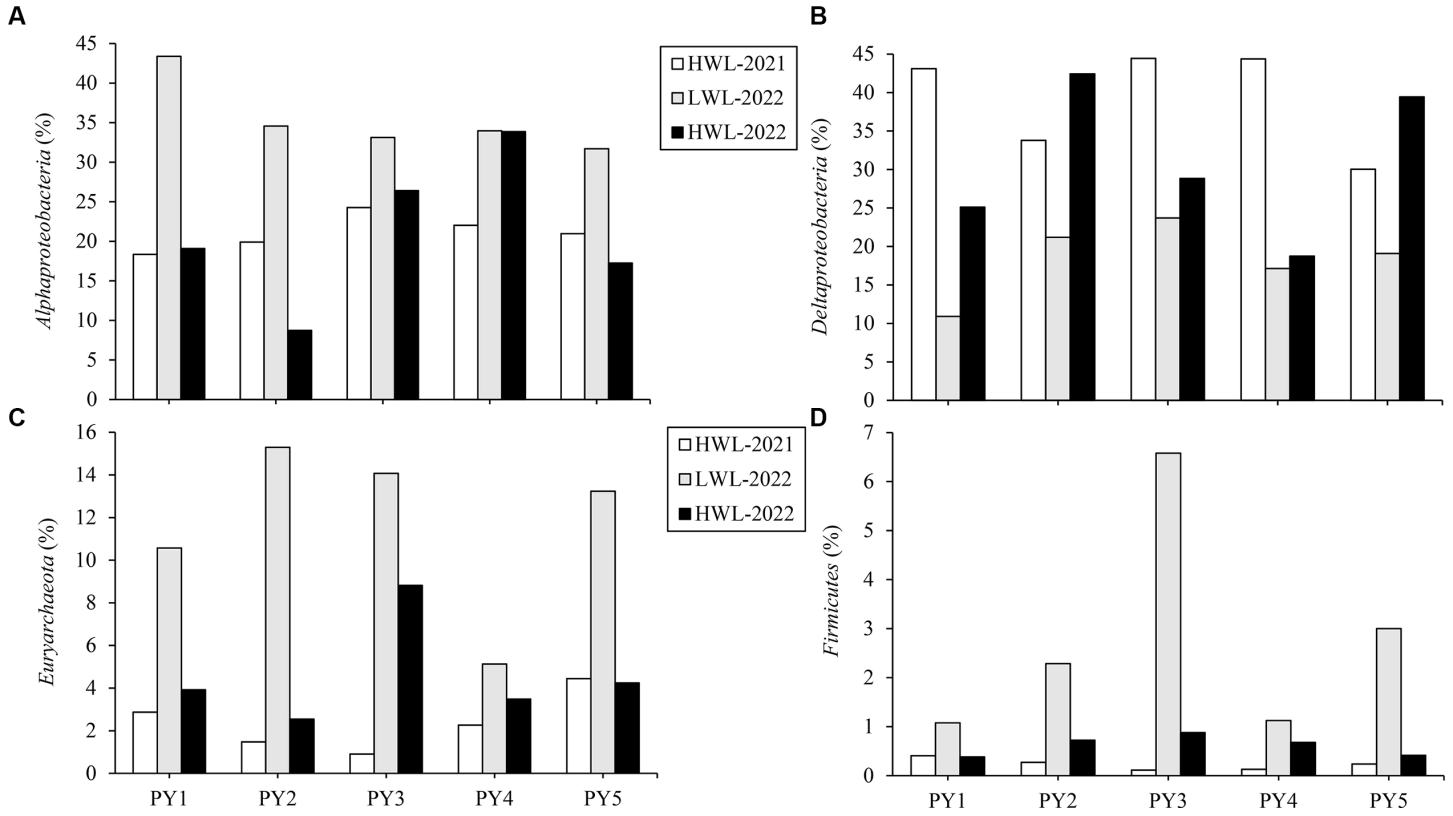
Relative abundance dynamics of **(A)** *Alphaproteobacteria*, **(B)** *Deltaproteobacteria*, **(C)** *Euryarchaeota*, and **(D)** *Firmicutes* at the five primary sites (PY1-PY5) among different water level phases.

At the five primary sites, some regular variation patterns of diazotrophic community composition with the water level phase transitions were observed at the phylum (or class) level (Figure 5). The relative abundance of *Alphaproteobacteria*, *Euryarchaeota* and *Firmicutes* was increased (by 8.9–25.0%, 2.9–13.8%, and 0.7–6.5%, respectively) in LWL-2022 compared with HWL-2021, whereas it was decreased (by 0.1–25.8%, 1.6–12.8%, and 0.4–5.7%, respectively) in HWL-2022 compared with LWL-2022. On the contrary, the relative abundance of *Deltaproteobacteria* decreased (by 11.0–32.2%) with the transition from the first high to low water level phases, while it increased (by 1.6–21.2%) with the transition from the low to second high water level phase. Interestingly, we found no regular variation patterns in the relative abundance of the above-mentioned diazotrophic phyla (or classes) at the five primary sites between the two high water level phases (Figure 5).

At the five primary sites, the regular changes of diazotrophic community composition with the water level phase transitions were also observed at the genus level (Supplementary Figure S5). The relative abundance of five genera (*Geobacter*, *Deferrisoma* and *Desulfuromonas* assigned to *Deltaproteobacteria*, and *Rivicola* and *Paraburkholderia* assigned to *Betaproteobacteria*) was decreased (by 6.9–23.4%, 1.6–3.2%, 1.8–3.6%, 1.0–3.1%, and 0.04–1.0%, respectively) in LWL-2022 compared with HWL-2021, whereas it was increased (by 1.8–23.9%, 0.2–1.0%, 0.1–1.1%, 0.4–3.9%, and 0.04–0.7%, respectively) in HWL-2022 compared with LWL-2022. Contrarily, the relative abundance of four genera (*Methylophilus* belonging to *Betaproteobacteria*, *Methanothrix*, and *Methanobacterium* belonging to *Euryarchaeota*, and *Clostridium* belonging to *Firmicutes*) increased (by 0.8–3.9%, 1.9–10.2%, 0.7–4.0%, and 0.3–3.4%, respectively) with the transition from the first high to low water level phases, while it decreased (by 0.6–4.4%, 2.3–10.2%, 0.1–3.5%, and 0.2–3.2%, respectively) with the transition from the low to second high water level phase. In addition, we found no regular variation patterns in the relative abundance of six of the nine above-mentioned diazotrophic genera (*Geobacter*, *Rivicola*, *Paraburkholderia*, *Methylophilus*, *Methanothrix*, and *Clostridium*) at the five primary sites between the two high water level phases (Supplementary Figure S5). Two diazotrophic genera (*Deferrisoma* and *Desulfuromonas*) were more abundant in HWL-2021 compared with HWL-2022 at the same sites (Supplementary Figure S5A), while one diazotrophic genus (*Methanobacterium*) was reversed (Supplementary Figure S5C).

At the five primary sites, the structure of diazotrophic communities at the OTU level was further compared among water level phases by using ANOSIM (Table 2), PerMANOVA (Table 2), and DCA methods (Supplementary Figure S6). Pairwise comparisons of ANOSIM and PerMANOVA both showed that the diazotrophic community structure in LWL-2022 was significantly different from that in HWL-2021 and HWL-2022 (Table 2). No significant difference in diazotrophic community structure was observed between the two high water level phases (Table 2). Supplementary Figure S6 shows that the diazotrophic communities in low (LWL-2022) and high (HWL-2021 and HWL-2022) water level phases were clearly separate from each other. The diazotrophic communities in the two high water level phases did not show a notable separation (Supplementary Figure S6). These findings suggested that the diazotrophic community structure was significantly affected by the declining water level, while the rising water level contributed to the recoveries of the diazotrophic community structure.

**Table 2:** Analysis of similarities (ANOSIM) and permutational multivariate analysis of variance (PerMANOVA) of diazotrophic community structure at the five primary sites (PY1-PY5) among different water level phases.

Comparison	ANOSIM		PerMANOVA	
	*R*	*p*	*R* ^2^	*p*
HWL-2021 versus LWL-2022	0.724	0.008	0.240	0.011
LWL-2022 versus HWL-2022	0.384	0.013	0.173	0.007
HWL-2021 versus HWL-2022	0.124	0.096	0.136	0.057

### 3.4 Changes in diazotrophs co-occurrence network

**Figure 6:**
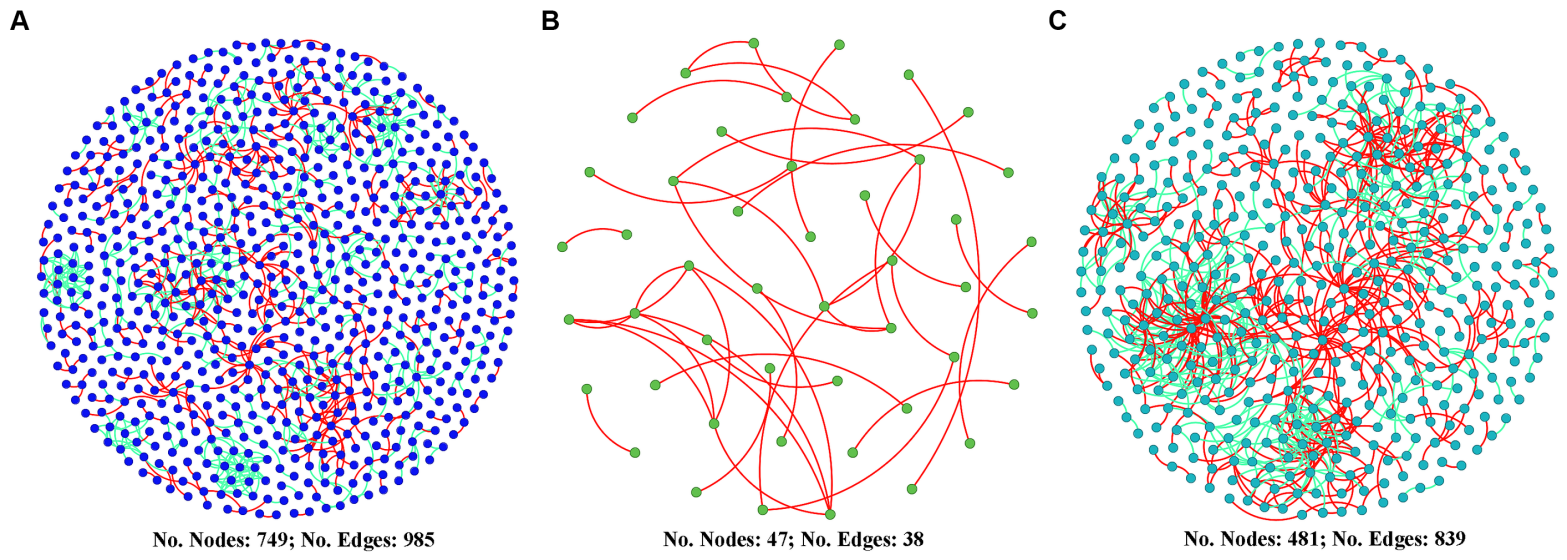
Diazotrophs co-occurrence networks for **(A)** HWL-2021, **(B)** LWL-2022, and **(C)** HWL-2022. Nodes represent OTUs. Red and green edges indicate positive and negative interactions, respectively.

Figure 6 shows that the co-occurrence network of the diazotrophic community was more complex in both the two high water level phases compared with the low water level phase. The number of nodes in HWL-2021 and HWL-2022 was 15.9 and 10.2 times that in LWL-2022, respectively. The number of edges in HWL-2021 and HWL-2022 was 25.9 and 22.1 times that in LWL-2022, respectively. Compared to LWL-2022, the average degree, average path distance, connectedness and number of modules were higher in HWL-2021 and HWL-2022 (Supplementary Table S3). Moreover, the numbers of putative keystone taxa of the diazotrophs network was higher in HWL-2021 and HWL-2022 than in LWL-2022 (Supplementary Figure S7). For HWL-2021, fifteen module hubs (two *Alphaproteobacteria*, seven *Gammaproteobacteria*, three *Deltaproteobacteria*, one *Kiritimatiellaeota*, one *Euryarchaeota*, and one *Nitrospirae*) were detected in the network (Supplementary Figure S7). For LWL-2022, no network hub, module hub or connector was detected in the network (Supplementary Figure S7A). For HWL-2022, thirteen module hubs (four *Alphaproteobacteria*, three *Betaproteobacteria*, one *Gammaproteobacteria*, and five *Deltaproteobacteria*) and three connectors (one *Alphaproteobacteria*, one *Betaproteobacteria*, and one *Gammaproteobacteria*) were detected in the network (Supplementary Figure S7). These results indicated that the diazotrophs co-occurrence network was disrupted by the declining water level, but it was strengthened by the rising water level.

### 3.5 Correlations between diazotrophic community and environmental factors

Six of the ten most abundant diazotrophic phyla (average relative abundance >1%) were significantly (*p* < 0.05) correlated with certain sediment properties (Table 3). *Euryarchaeota* and *Firmicutes* were significantly and negatively correlated with TN, TP and N:P molar ratio. *Nitrospirae* and *Spirochaetes* were significantly and positively related to TP and pH, respectively. *Alphaproteobacteria* was significantly and negatively correlated with TN and N:P molar ratio, whereas *Deltaproteobacter* was positively correlated.

**Table 3:** Pearson correlation coefficients of the relative abundance of the abundant diazotrophic phyla (average relative abundance >1%) with sediment properties.

	pH	EC	TN	TP	OM	N:P
*Bacteroidetes*	0.26	−0.006	0.14	0.11	0.20	0.14
*Cyanobacteria*	0.02	−0.40	0.004	−0.02	0.03	0.02
*Euryarchaeota*	0.07	0.28	***−0.74***	***−0.64***	−0.16	***−0.58***
*Firmicutes*	0.20	0.28	***−0.76***	***−0.68***	−0.11	***−0.55***
*Nitrospirae*	0.07	−0.15	0.28	**0.43**	−0.29	−0.02
*Spirochaetes*	**0.49**	−0.09	−0.06	0.15	0.01	−0.31
*Alphaproteobacteria*	0.38	0.20	**−0.49**	−0.21	−0.07	***−0.69***
*Betaproteobacteria*	−0.09	−0.39	0.20	0.09	0.21	0.25
*Deltaproteobacteria*	−0.36	−0.04	**0.50**	0.22	−0.19	***0.68***
*Gammaproteobacteria*	−0.35	0.03	0.36	0.29	0.17	0.31

The phylum Proteobacteria is represented by its four major subdivisions (*Alphaproteobacteria*, *Betaproteobacteria*, *Deltaproteobacteria*, and *Gammaproteobacteria*).

Bold indicates *p* < 0.05; bold italics indicate *p* < 0.01.

Significant (*p* < 0.05) correlations were also observed between nine of the fifteen most abundant diazotrophic genera (average relative abundance >1%) and certain sediment properties (Table 4). *Bradyrhizobium* belonging to *Alphaproteobacteria* and *Desulfuromonas* belonging to *Deltaproteobacteria* were correlated significantly with pH. *Rhodopseudomonas* assigned to *Alphaproteobacteria*, *Methylophilus* assigned to *Betaproteobacteria*, *Desulfomonile* assigned to *Deltaproteobacteria* and *Methanothrix* assigned to *Euryarchaeota* were significantly and negatively correlated with TN, whereas *Rivicola* assigned to *Betaproteobacteria* and *Geobacter*, *Deferrisoma*, and *Desulfuromonas* assigned to *Deltaproteobacteria* were positively correlated. *Methylophilus*, *Desulfomonile*, and *Methanothrix* were significantly and negatively related to TP. *Bradyrhizobium*, *Rhodopseudomonas*, *Methylophilus*, and *Methanothrix* were significantly and negatively correlated with N:P molar ratio, whereas *Rivicola*, *Geobacter*, *Deferrisoma*, and *Desulfuromonas* were positively correlated.

**Table 4:** Pearson correlation coefficients of the relative abundance of the abundant diazotrophic genera (average relative abundance >1%) with sediment properties.

	pH	EC	TN	TP	OM	N:P
***Alphaproteobacteria***						
*Bradyrhizobium*	**0.45**	0.05	−0.27	0.01	−0.08	**−0.52**
*Methylocystis*	−0.20	0.32	−0.38	−0.29	−0.03	−0.40
*Rhodopseudomonas*	0.11	0.04	**−0.49**	−0.31	0.12	**−0.51**
***Betaproteobacteria***						
*Methylophilus*	−0.15	0.38	**−0.52**	**−0.44**	0.10	**−0.45**
*Rivicola*	−0.30	−0.17	**0.48**	0.31	0.07	**0.53**
*Thiobacillus*	−0.29	0.004	0.13	0.08	−0.32	0.14
***Deltaproteobacteria***						
*Geobacter*	−0.32	−0.13	***0.58***	0.33	−0.07	***0.70***
*Deferrisoma*	−0.29	0.01	**0.48**	0.20	−0.26	***0.67***
*Desulfuromonas*	**−0.43**	0.05	**0.49**	0.36	−0.16	**0.48**
*Desulfomonile*	−0.17	0.04	***−0.54***	**−0.47**	−0.30	−0.41
***Gammaproteobacteria***						
*Methylobacter*	−0.31	−0.02	0.38	0.36	0.15	0.26
*Methylomicrobium*	−0.27	0.06	0.22	0.13	0.27	0.24
*Methylomonas*	−0.34	−0.04	0.40	0.28	0.15	0.40
***Cyanobacteria***						
*Anabaena*	0.008	−0.40	0.004	−0.02	0.01	0.02
***Euryarchaeota***						
*Methanothrix*	−0.10	0.29	***−0.65***	***−0.56***	−0.17	**−0.52**

Bold indicates *p* < 0.05; bold italics indicate *p* < 0.01.

**Figure 7:**
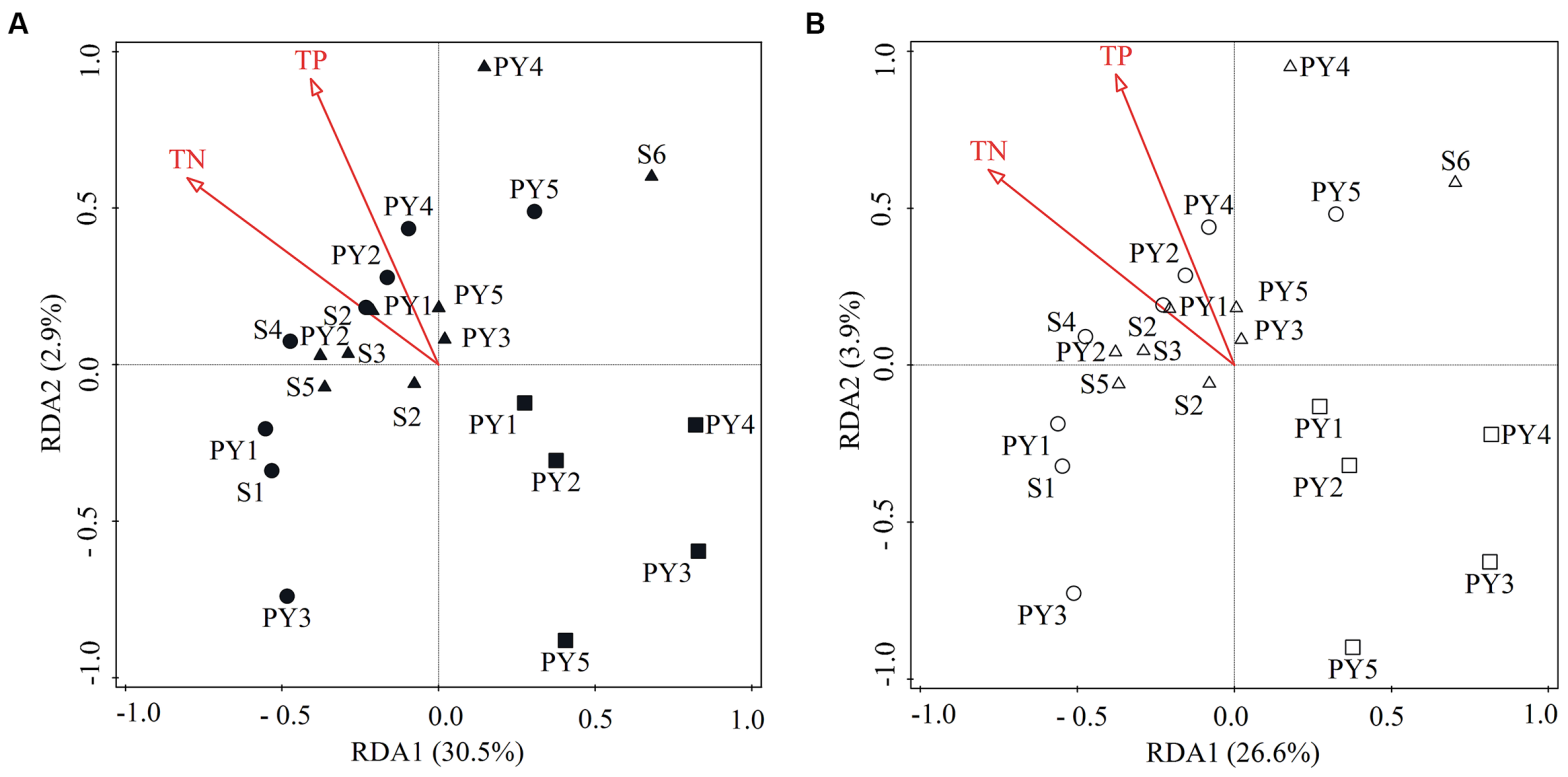
RDA ordination plots showing the relationship between the significant environmental variables (*p* < 0.05) and the diazotrophic community composition at **(A)** phylum and **(B)** genus level. Circle, square and triangle symbols indicate the samples from HWL-2021, LWL-2022, and HWL-2022, respectively. The phylum *Proteobacteria* is represented by its classes.

Results of RDA illustrated that TN and TP were the most significant variables (Monte Carlo test, *p* < 0.05) in the diazotrophic community composition, explaining 33.4 and 30.5% of the changes at the phylum (Figure 7A) and genus level (Figure 7B), respectively.

## 4 Discussion

### 4.1 Composition of diazotrophic communities in the sediments of Poyang Lake

In this study, sequencing of *nifH* gene revealed that diazotrophs in the sediments of Poyang Lake mainly belonged to *Proteobacteria*, *Euryarchaeota*, *Cyanobacteria*, *Bacteroidetes*, *Firmicutes*, *Nitrospirae*, and *Spirochaetes* (Figure 3), which have been previously reported to be among the diazotrophic community from Lake Taihu, Lake Fuxian, Dongzhen Reservoir (China) and Pacific Ocean (Yu et al., 2014; Kapili et al., 2020; Wang et al., 2020; Tian et al., 2021a). Consistent with the report on Lake Taihu (Tian et al., 2021a), *Proteobacteria* was the predominant phylum in the diazotrophic communities from the sediments in this study. *Proteobacteria* has also been found to be the most dominant group in sediment microbial communities in Poyang Lake (Ni et al., 2016). Consistent with the report on two Chinese freshwater lakes (Lake Taihu and Dajiuhu) (Xu et al., 2019; Tian et al., 2021a), *Alphaproteobacteria*, *Betaproteobacteria*, *Deltaproteobacteria* and *Gammaproteobacteria* were the four dominant classes within the members of *Proteobacteria* found in the sediment diazotrophic communities of Poyang Lake (Supplementary Figure S4).

Diazotrophs are generally clustered into four basic groups based on phylogenetic distribution of *nifH* genes, including Cluster I-IV (Chien and Zinder, 1996). In this study, we detected Cluster I, Cluster II and Cluster III diazotrophs in the sediments of Poyang Lake (Figure 4). The members of Cluster I and Cluster III are frequently found in freshwater habitats (Wang et al., 2020; Tian et al., 2021a,b). The composition of diazotrophic communities in the sediments of Lake Taihu have been shown to mainly consist of Clusters I and III (Tian et al., 2021a,b), which is similar to our data in Poyang Lake (Figure 4). Different from the findings in two Chinese freshwater lakes (Lake Fuxian and Lake Taihu) (Wang et al., 2020; Tian et al., 2021a,b), we found that one OTU (OTU16), belonging to *Afipia*, was assigned to Cluster II in the present study. *Afipia* has been reported to be present in the soybean root nodules (Bromfield et al., 2017).

### 4.2 Diazotrophic community response to WLFs

In this study, the results of ANOSIM, PerMANOVA and DCA suggested that the declining water level resulted in the changes of diazotrophic community composition, and the rising water level contributed to the recoveries of diazotrophic community structure. It has been reported that WLFs have effect on both the structure and functioning of lake ecosystems (Coops et al., 2003; Evtimova and Donohue, 2016). Previous studies have shown the variations of macrophyte communities, benthic invertebrates, assemblage structure and gross primary productivity of phytoplankton, and microbial community structure and function with WLFs in natural lakes (Coops et al., 2003; Liu et al., 2015; Evtimova and Donohue, 2016; Qian et al., 2016; Liu et al., 2019; Ren et al., 2019b; Wang S. Y. et al., 2021). However, the information on the effect of WLFs on the microbial community related to nitrogen cycle is scarce. In addition, no regular variation patterns of both the alpha diversity indices and *nifH* gene copies of diazotrophs were observed with the water level phase transitions in the present study. These results indicated that WLFs had no effect on the alpha diversity and abundance of diazotrophs. This is inconsistent with the previous report that alpha diversity of planktonic bacteria in dry-season differs from that in wet-season in Poyang Lake (Ren et al., 2019b). However, a previous study has found that the benthic-bacterial cell density is not affected by WLFs in its experimental wetlands (Ishida et al., 2006). It has also been reported that microbial biomass and Shannon diversity in the water are affected by groundwater table fluctuations, while those in the river bed sediments remain stable (Zhou et al., 2012). It was possible that WLFs had no effect on microorganisms inhabited the sediments of freshwater bodies in terms of alpha diversity and abundance.

The co-occurrence network of diazotrophs was more complex in both the two high water level phases compared with the low water level phase in our study (Figure 6). Moreover, no putative keystone OTU was detected in the low water level phase, while 15 and 16 putative keystone OTUs were detected in the first and second high water level phase, respectively (Supplementary Figure S7). These findings suggested that the diazotrophs network was disrupted by the declining water level, but it was strengthened by the rising water level. Compared to LWL-2022, the levels of TN and TP were higher in HWL-2021 and HWL-2022. A previous study has reported that the members of the diazotrophic community are more associated with each other in the nutrient-rich soil, whereas diazotrophs have less cooperative and competitive interactions in the resource-poor soil (Wang H. et al., 2021), suggesting that an ecosystem with more available nutrients tends to maintain a complex network of diazotrophs. A previous study has also suggested that both low N and high P input promote network association among soil diazotrophs in an Inner Mongolia steppe (China) (Wang et al., 2023). Thus, we speculated that the content variations of TN and TP in consequence of the WLFs might result in the changes of diazotrophs co-occurrence pattern in the sediments. However, the speculation needed to be further confirmed.

### 4.3 Environmental factors regulating diazotrophic community composition

In our study, the levels of TN and TP were decreased from the first high to low water level phase, and increased from the low to second high water level phase (Figures 2A,B). Previous studies have also suggested that the WLFs play an important role in sediment N and P dynamics in freshwater bodies (Ni et al., 2015; Coppens et al., 2016; Yu et al., 2020). Nutrients may be easier to be released from sediment to water in the low water level phase in lake (Liu et al., 2016; Geng et al., 2022; Ma et al., 2023), and thus lead to the reduction of TN and TP contents in sediments. In wet seasons, a high loading of nutrients from the five rivers (Gan, Fu, Xin, Rao and Xiu Rivers) is a major source of N and P in Poyang Lake (Dai and Hu, 2019). It might be speculated that the high loading of nutrients may result in increasing of sediment N and P contents. In addition, the N:P molar ratio was also decreased from the first high to low water level phase, and increased from the low to second high water level phase (Figure 2C). This finding might imply that the molar content of N release was higher than P release from sediment to water in the low water level phase, and the molar content increasing of sediment N was higher than sediment P in the high water level phase.

It has been previously reported that environmental factors play an important role in structuring the diazotrophic community (Weber et al., 2017; Luo et al., 2021; Jabir et al., 2021a; Tian et al., 2021a; Jabir et al., 2021b). Results of RDA indicated that TN and TP were the most significant variables explaining the variation of the diazotrophic community composition (Figure 7). Similarly, the concentrations of TN and TP have also been reported to be significantly related to the variation of the diazotrophic community structure in Lake Fuxian and an Arctic fjord (Kongsfjorden) (Wang et al., 2020; Jabir et al., 2021b). In the present study, the diazotrophic community composition, TN and TP varied with the WLFs. Hence, we speculated that the WLFs might influence sediment N and P contents, and in turn influence the diazotrophic community composition.

In this work, eight of the fifteen most abundant diazotrophic genera were significantly correlated with TN (Table 4). Among them, *Geobacter* and *Desulfuromonas* were positively associated with total nitrogen, and *Desulfomonile*, *Methylophilus*, and *Rhodopseudomonas* were negatively associated with total nitrogen. Fan et al. (2018) have reported that total nitrogen significantly and positively affect the abundance of *Geobacter* in the sediments of a Chinese freshwater lake (Chaohu Lake). *Geobacter* species have also been previously found to be stimulated by long-term N fertilization in paddy soils (Ding et al., 2015). *Desulfuromonas* has been previously reported to be significantly and positively associated with nitrogen densities in the saltmarshes soils within the Sydney Olympic Park (Australia) (Santini et al., 2019), which is similar to our data in the sediments of Poyang Lake. A previous study has shown that lower N levels may contribute to an increase in the amount of *Desulfomonile* in mangrove swamps in the North Beibu Gulf (China) (Li et al., 2022). FmdC, an outer-membrane porin in a *Methylophilus* strain, can be induced by short-chain amides and urea, and transport these nitrogen sources under conditions where they are present at very low concentration (Mills et al., 1997). In contrast to our findings, a previous work has shown that *Rhodopseudomonas* is stimulated by the nitrogen applications in *Malus sieversii* rhizosphere soils (Zhang et al., 2022). In the present study, three of the fifteen most abundant diazotrophic genera were significantly correlated with TP (Table 4). Among them, *Desulfomonile* has been reported to be more abundant at lower concentrations of total phosphorus in mangrove swamps in the North Beibu Gulf (China) (Li et al., 2022), which is consistent with our study. In addition, the diazotrophic phylum *Nitrospirae* was significantly and positively associated with TP in our study (Table 3). Similarly, Huang et al. (2017) have found that *Nitrospirae* is more abundant in the sediments of the moderately eutrophic lakes compared to the lightly eutrophic and moderately trophic lakes in China.

## 5 Conclusion

Our study represents the first to investigate the response of sediment diazotrophic communities in the floodplain lake to water level fluctuations (WLFs). The diazotrophic community structure was significantly affected by the declining water level, while the rising water level contributed to the recoveries of the diazotrophic community structure. The relative abundance of *Alphaproteobacteria*, *Euryarchaeota* and *Firmicutes* was increased with the transition from the high to low water level phases, whereas it was decreased with the transition from the low to high water level phase. WLFs had no effect on the alpha diversity and abundance of the diazotrophic community. It is noteworthy that the diazotrophs co-occurrence network was disrupted by the declining water level, but it was strengthened by the rising water level. Our results further demonstrated that the variation of the diazotrophic community composition was mostly related to sediment TN and TP. The data obtained in this study will expand our understanding of the ecological impacts of WLFs and the nitrogen fixation process of the floodplain lake.

## Data availability statement

The datasets presented in this study can be found in online repositories. The names of the repository/repositories and accession number(s) can be found in the article/Supplementary material.

## Author contributions

QW: Conceptualization, Formal analysis, Writing – original draft, Writing – review & editing. FW: Formal analysis, Writing – review & editing. YC: Conceptualization, Investigation, Writing – original draft. WZ: Investigation, Writing – original draft. ZZ: Investigation, Writing – original draft.

## Footnotes

^1^http://www.jxssw.gov.cn/

^2^http://ccb.jhu.edu/software/FLASH

^3^http://drive5.com/uparse/

^4^http://ieg4.rccc.ou.edu/mena/
