# Supplementary material for: Diazotrophic community in the sediments of Poyang Lake in response to water level fluctuations
Source: Front Microbiol. 2024 Feb 2;15:1324313. doi: 10.3389/fmicb.2024.1324313 (PMC10869460; doi:10.3389/fmicb.2024.1324313)

***Supplementary Material***

**Table S1** The diversity index and abundance of diazotrophs in the three water level phases in Poyang Lake.

|  | Water level phases | | |
| --- | --- | --- | --- |
|  | HWL-2021 | LWL-2022 | HWL-2022 |
| OTU Richness | 2261 ± 883 | 2089 ± 841 | 2402 ± 1344 |
| Chao | 2685 ± 1091 | 2550 ± 987 | 2810 ± 1610 |
| Shannon | 6.62 ± 0.51 | 6.46 ± 0.53 | 6.38 ± 0.92 |
| *nifH* gene (10^8^ copies g^−1^) | 2.80 ± 1.38 | 4.68 ± 3.21 | 6.63 ± 4.17 |

Note: Values are means ± standard deviations.

**Table S2** Pearson correlation coefficients of the alpha diversity indices and abundance of diazotrophs with sediment properties.

|  | pH | EC | TN | TP | OM | N:P |
| --- | --- | --- | --- | --- | --- | --- |
| OTU Richness | -0.37 | -0.17 | 0.18 | 0.12 | 0.15 | 0.19 |
| Chao | -0.38 | -0.15 | 0.13 | 0.07 | 0.13 | 0.16 |
| Shannon | ***-*0.46** | -0.09 | 0.16 | 0.06 | 0.06 | 0.22 |
| *nifH* gene abundance | -0.16 | -0.26 | 0.18 | 0.19 | **0.44** | 0.10 |

Note: Bold indicates *P* < 0.05.

**Table S3** Topological properties of the co-occurrence network of diazotrophs in the three water level phases in Poyang Lake.

|  | Water level phases | | |
| --- | --- | --- | --- |
|  | HWL-2021 | LWL-2022 | HWL-2022 |
| Average degree (avgK) | 2.630 | 1.617 | 3.489 |
| Average path distance (GD) | 10.132 | 1.000 | 7.031 |
| Connectedness | 0.182 | 0.035 | 0.661 |
| Modularity (no. of modules) | 0.939 (88) | 0.882 (20) | 0.809 (44) |

**Figure S1.** Rarefaction curves based on (**A**) OTUs and (**B**) Shannon index for all diazotrophic communities.


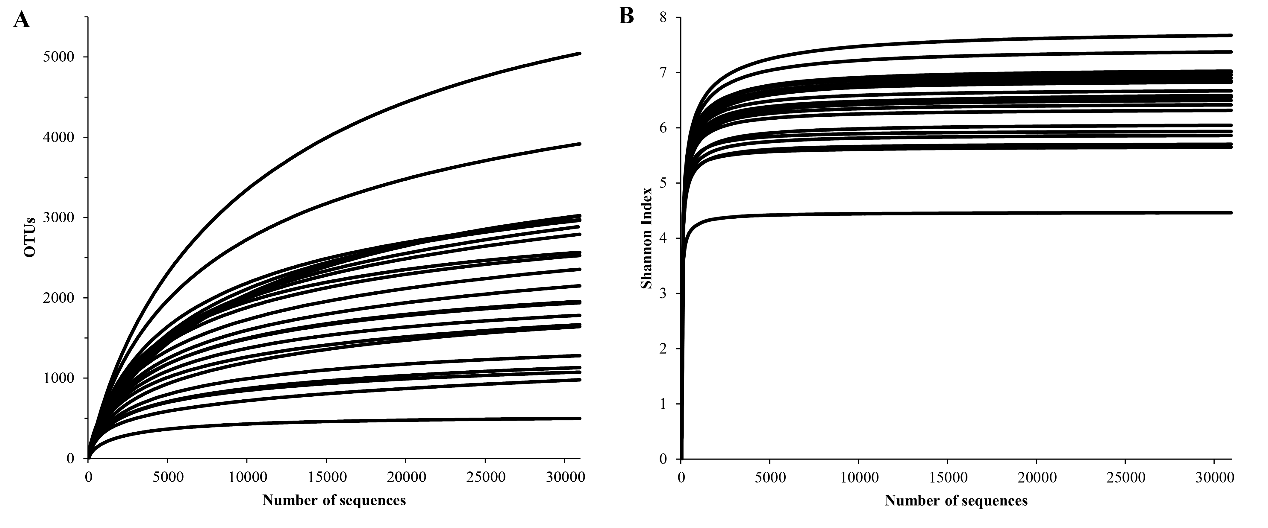


**Figure S2.** Dynamics of (**A**) OTU Richness, (**B**) Chao richness estimators, (**C**) Shannon diversity index and (**D**) *nifH* gene copies at the five primary sites (PY1-PY5) among different water level phases.


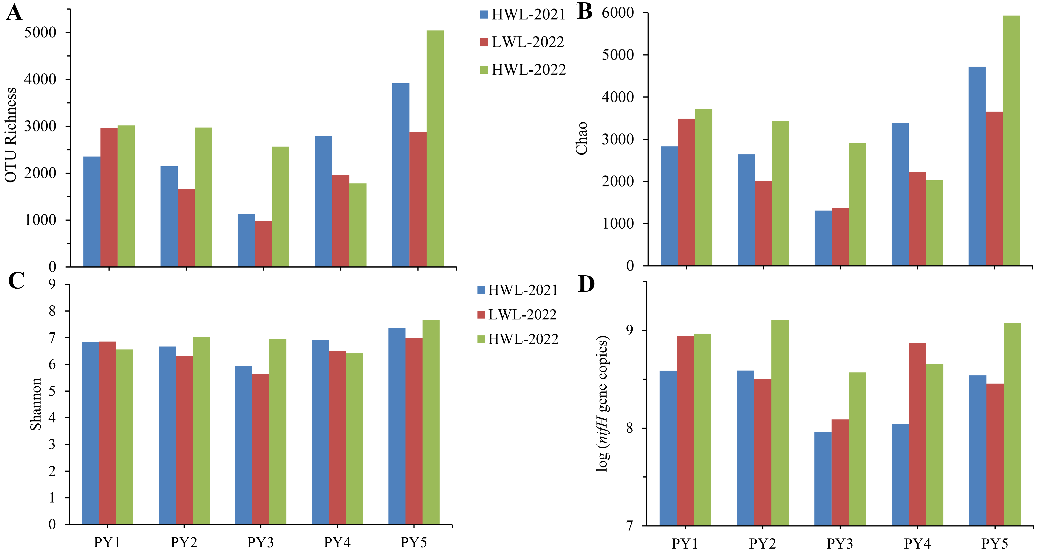


**Figure S3.** Relationships between (**A**) OTU Richness, (**B**) Chao richness estimators and (**C**) Shannon diversity index and *nifH* gene copies. The dependent and independent variables were log (*x*+1) transformed.


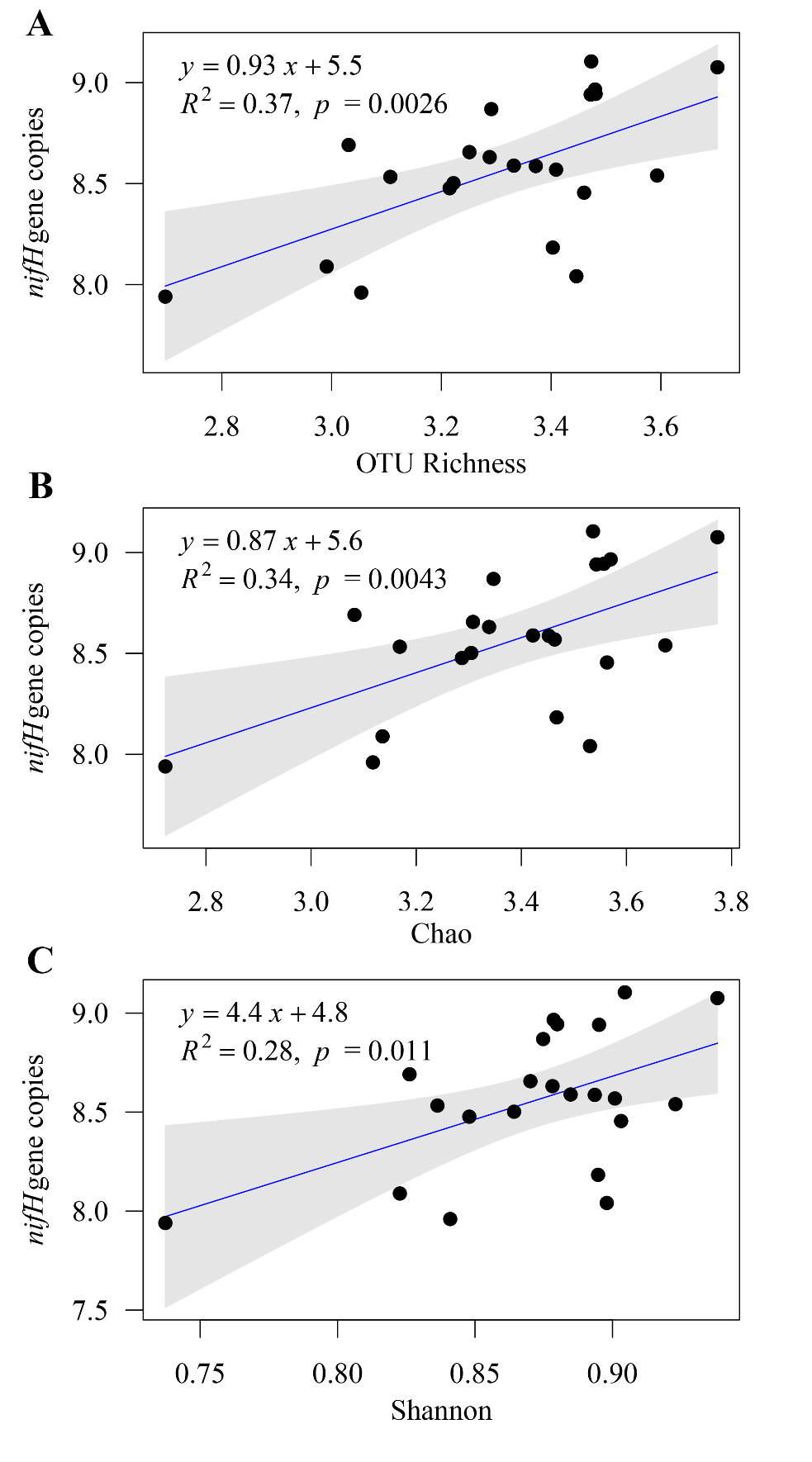


**Figure S4.** Phylogenetic composition of *Proteobacteria* found in the three water level phases.

**
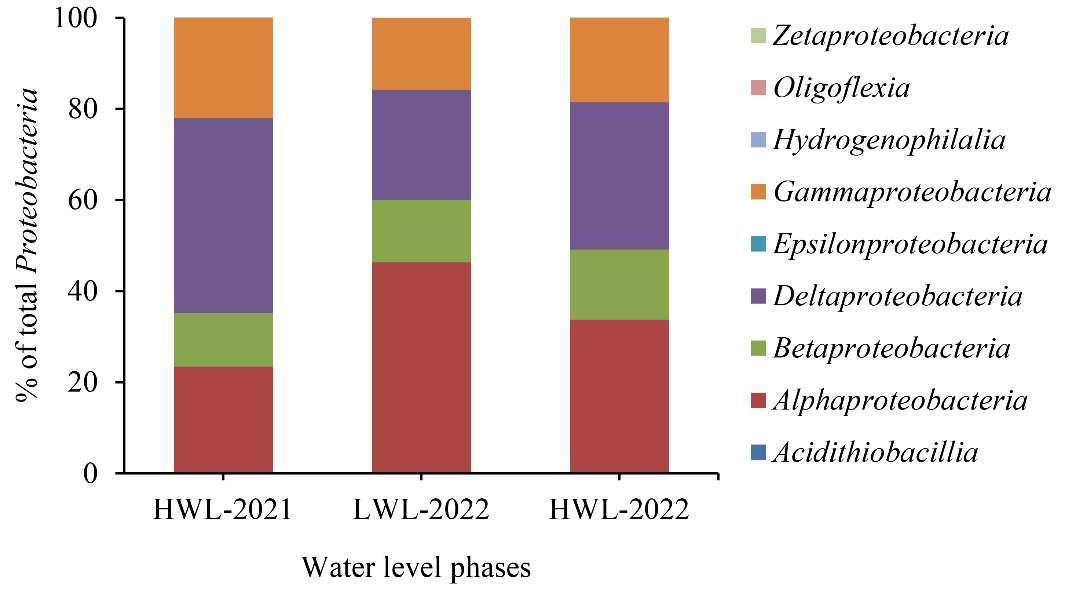
**

**
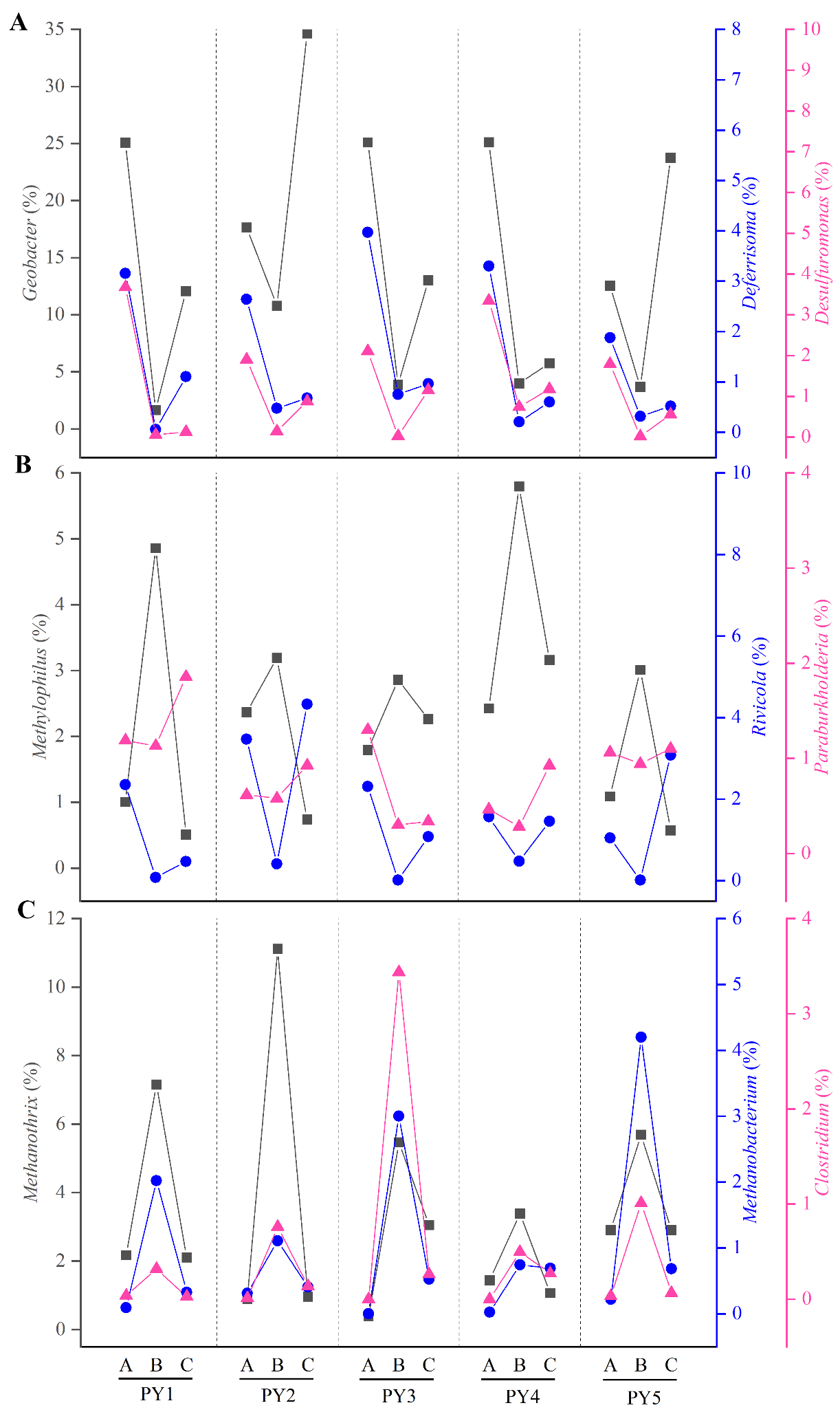
Figure S5.** Relative abundance of the genera selected to compare the composition of diazotrophic communities at the five primary sites (PY1-PY5) among different water level phases. HWL-2021, LWL-2022 and HWL-2022 are represented by A, B and C, respectively. (**A**) Three genera of *Deltaproteobacteria*. (**B**) Three genera of *Betaproteobacteria*. (**C**) Two genera of *Euryarchaeota* and one genus of *Firmicutes* (*Clostridium*).

**Figure S6.** DCA ordination plots of the diazotrophic OTUs at the five primary sites (PY1-PY5). Circle, square and triangle symbols indicate the samples from HWL-2021, LWL-2022 and HWL-2022, respectively.


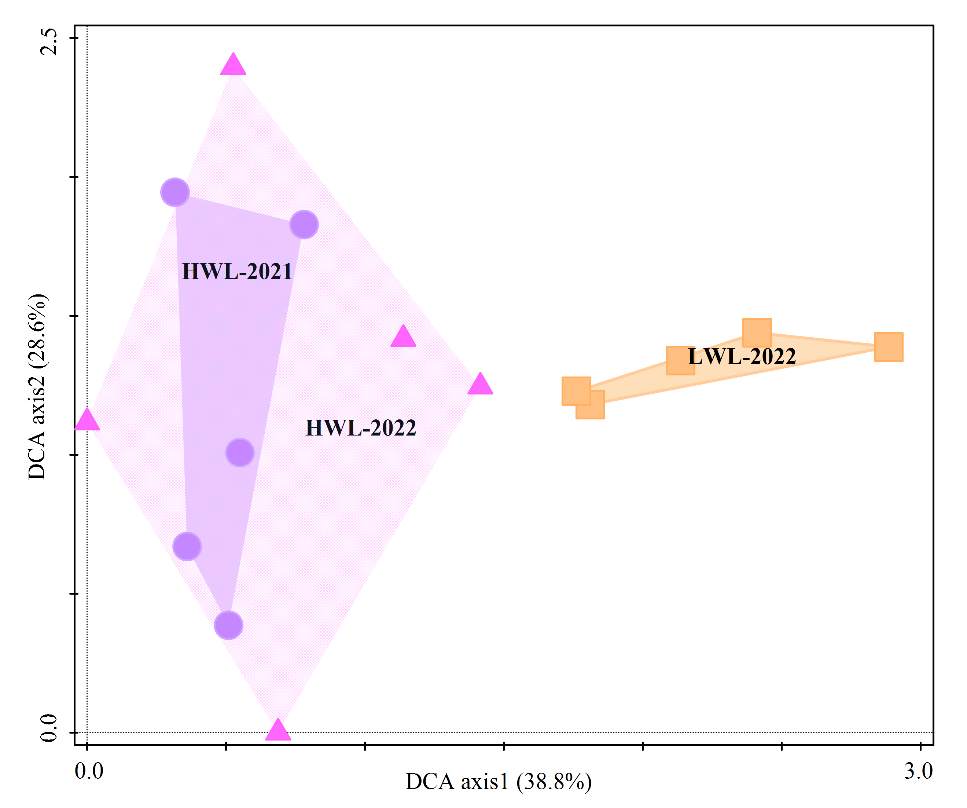


**Figure S7.** (**A**) Zi-Pi (Zi: within-module connectivity and Pi: among-module connectivity) plot for recognition of putative keystone taxa. Module hubs have Zi > 2.5 and Pi < 0.62, and connectors have Zi < 2.5 and Pi > 0.62. (**B**) Number and taxonomic classification of putative keystone OTUs.


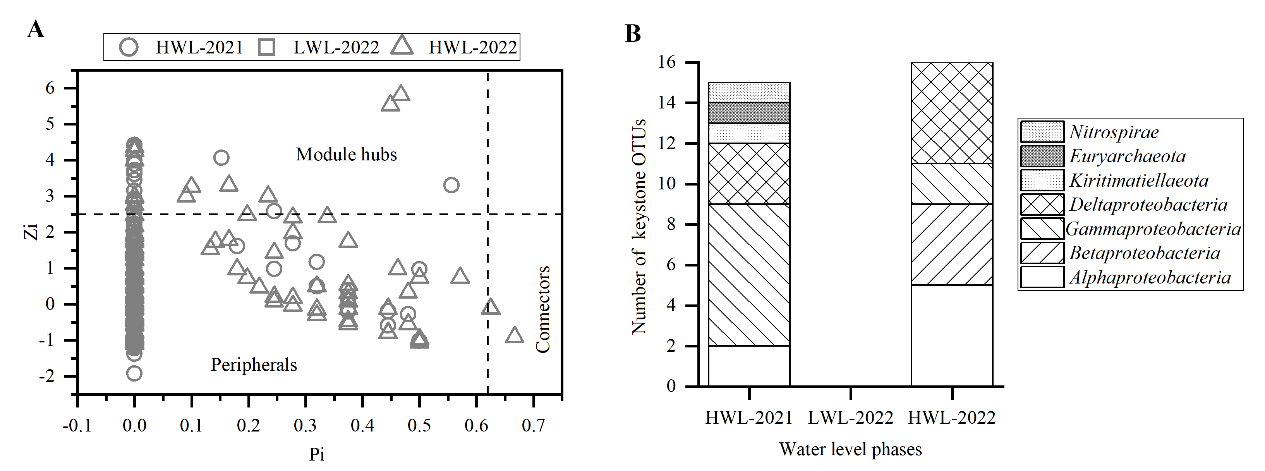

Supplement: Supplementary file 1 [file Data_Sheet_1.docx]
